# Patient participation in electronic nursing documentation: An interview study among home‐care patients

**DOI:** 10.1111/hex.13492

**Published:** 2022-04-05

**Authors:** Kim De Groot, Judith Douma, Wolter Paans, Anneke L. Francke

**Affiliations:** ^1^ Departement of Nursing Care and Elderly Care Netherlands Institute for Health Services Research (Nivel) Utrecht The Netherlands; ^2^ Thebe Wijkverpleging (Home‐Care Organisation) Tilburg The Netherlands; ^3^ Nursing Science, Programme in Clinical Health Sciences, University Medical Centre Utrecht Utrecht University Utrecht The Netherlands; ^4^ Research Group Nursing Diagnostics, Research Group Nursing Diagnostics, School of Nursing Hanze University of Applied Sciences Groningen The Netherlands; ^5^ Department of Critical Care University Medical Centre Groningen Groningen The Netherlands; ^6^ Department of Public and Occupational Health, Amsterdam Public Health Research Institute, Amsterdam University Medical Centre Vrije Universiteit Amsterdam Amsterdam The Netherlands

**Keywords:** electronic health record, home care, nursing documentation, patient involvement, patient participation

## Abstract

**Background:**

Patients are increasingly expected to take an active role in their own care. Participation in nursing documentation can support patients to take this active role since it provides opportunities to express care needs and preferences. Yet, patient participation in electronic nursing documentation is not self‐evident.

**Objective:**

To explore how home‐care patients perceive their participation in electronic nursing documentation.

**Methods:**

Semi‐structured interviews were conducted with 21 home‐care patients. Interview transcripts were analysed in an iterative process based on the principles of reflexive inductive thematic analysis.

**Results:**

We identified a typology with four patient types: ‘high need, high ability’, ‘high need, low ability’, ‘low need, high ability’ and ‘low need, low ability’. Several patients felt a need for participation because of their personal interest in health information. Others did not feel such a need since they trusted nurses to document the information that is important. Patients' ability to participate increased when they could read the documentation and when nurses helped them by talking about the documentation. Barriers to patients' ability to participate were having no electronic devices or lacking digital skills, a lack of support from nurses and the poor usability of electronic patient portals.

**Conclusion:**

Patient participation in electronic nursing documentation varies between patients since home‐care patients differ in their need and ability to participate. Nurses should tailor their encouragement of patient participation to individual patients' needs and abilities. Furthermore, they should be aware of their own role and help patients to participate in the documentation.

**Patient or Public Contribution:**

Home‐care patients were involved in the interviews.

## 1 INTRODUCTION

In today's healthcare system, value is attached to patient participation. We define patient participation, in line with the definition of Castro et al.,^1^ as: ‘the individual's engagement in the decision making about his care through a dialogue attuned to his preferences, potential and a combination of his experiential and the professional's expert knowledge’. Given the current attention in healthcare for patient participation, patients are expected to take an active role in their own care. When taking such an active role, this will enhance shared decision‐making of patients and professionals involved about the care, and about how the needs and preferences of the patient have to be met.^2^, ^3^ A growing body of evidence demonstrates that both patient participation and shared decision‐making can contribute to improved quality of life, better health outcomes and greater patient satisfaction.^4^, ^5^, ^6^, ^7^

Patient participation and shared decision‐making are easier to achieve when patients also participate in the care‐related documentation in their individual electronic health record.^8^, ^9^, ^10^ With patient participation in nursing documentation we mean in this paper that a patient is consulted by nurses during the documentation process, is involved in making the individual care plan, is involved in the actual documentation in the electronic health record, and/or reviews, corrects and supplements the information documented. Patients will be better able to express their preferences about nursing care when nurses ask them which information they believe is important to document,^11^ leading to care plans tailored to the needs of patients.^12^ Particularly when electronic health records are linked with electronic patient portals, this provides opportunities for patients to have control over their care and related decision‐making.^13^, ^14^, ^15^, ^16^, ^17^ Electronic patient portals are applications maintained by healthcare organizations that allow patients independent access to their individual health record.^16^

Yet patient participation in electronic nursing documentation is not self‐evident. In a qualitative interview study, Dutch community nurses mentioned various barriers for patient participation in documentation, such as poor internet connections, technical failures in the electronic health records and time pressure.^18^ These barriers made that nurses not always documented in the presence of patients and thereby limited patients to participate in the documentation.^18^ In addition, a focus group with four Dutch patients and four family caregivers indicated that patients often felt not involved in nursing documentation, for example, because documentation often occurred out of their sight, and they often could not access their individual health record.^19^ However, this focus group only involved patients who all were interested in nursing documentation, and more in‐depth insight was needed into patients' experiences and perspectives regarding participation in electronic nursing documentation. We chose to focus on the home‐care setting, given that in the Dutch context this setting is in a leading position in this regard: In 2019 81% of nurses in home care used electronic patient portals, compared to 42% of nurses working in general practitioner practices and 67% of the hospital nurses.^17^ Moreover, home‐care patients often have a long‐lasting care relationship with community nurses,^20^ which might make their participation in nursing documentation more important and feasible compared to patients in acute or short‐during care settings.

Therefore the following research questions are addressed in this article:

1. What are the reasons why home‐care patients do or do not participate in electronic nursing documentation?
2. In what ways do home‐care patients participate in electronic nursing documentation?

## 2 METHODS

### 2.1 Design

A qualitative descriptive design was used, based on principles of reflexive inductive thematic analysis.^21^, ^22^ We conducted semi‐structured interviews with 21 home‐care patients.

### 2.2 Sampling and setting

This study was conducted in the home‐care setting in the Netherlands. In this country, home care is provided by registered nurses and certified nursing assistants and involves personal physical care, technical care, preventive care and psychosocial care.^23^

We used purposive sampling to recruit patients who met the following inclusion criteria: (1) receiving home care from a care organization that uses electronic health records; (2) Dutch‐speaking and (3) having no severe cognitive impairments.

The participants were recruited with the assistance of community nurses from the professional networks of two of the authors (K. D. G. and J. D.) who both combined their position as researchers with their employment as community nurses. No patients with which these authors had a nurse–patient relationship were interviewed by these authors.

The authors instructed nurses to search for patients meeting the inclusion criteria, but also with some variation in age, gender, educational level, cultural background, living alone or with a spouse and the type of home care used. This variation was pursued because we assumed that these background characteristics were associated with the perspectives and experiences regarding patient participation in nursing documentation.

The community nurses provided the patients with an information letter and passed on the phone numbers of the patients who were willing to take part in the study. Recruitment stopped when analyses of the last two interviews showed that data saturation had been reached.

### 2.3 Data collection

Twenty‐one interviews were conducted between April 2019 and April 2021. Each interview was conducted by either one author (K. D. G. or J. D.) or by pairs of nursing students who were trained in interview techniques. The authors used insights from prior research,^19^ relevant Dutch legislation^24^ and a Dutch professional guideline on nursing documentation^25^ to create the interview guide that structured the interviews (Table 1). We refined questions of the interview guide during the cyclic process of data collection and analysis, to ensure that we were given in‐depth information needed to answer the research questions.

**Table 1:** Interview guide

1. Can you tell me what kind of home care you receive from the community nurses and how long you have been receiving this care?
2. Do you participate in what the community nurses document about the care you receive? If not, why not and how do you experience this? If so, how do you participate and how do you experience this? Which parts of the documentation can you participate in?
3. How important do you perceive participation in nursing documentation? If not, why isn't this important to you? If so, why do you think this is important?
4. Do you use an electronic patient portal? If not, why not? If so, what is your experience of this?
5. Can your family caregivers participate in nursing documentation? What do you think about that?
6. How do you think it could be made easier for you as a patient to participate in nursing documentation?

Initially, we aimed to conduct all interviews face‐to‐face at patients' homes or another place convenient for patients. We were able to do this for the first 13 interviews. However, because of the COVID‐19 pandemic, the last eight interviews were conducted over the phone. All interviews were audio‐recorded. In three interviews, the patient's spouse attended the interview and, although the interview was focused on the patient, the spouse sometimes gave a reaction as well. In the analysis, we only included the patient's remarks and not those of the spouse because the interview was focused on the patient.

### 2.4 Data analysis

The recordings of the interviews were transcribed verbatim. The interview transcripts were analysed in an iterative cyclic process of ‘data collection–data analysis–additional data collection’. This process implied that shortly after conducting 2–4 interviews, the transcripts were analysed and findings from this interim analysis steered questions for the following interviews. The cyclic process of data collection and data analysis continued until data saturation was reached, which was indicated by the fact that the analysis of the last two interviews produced no new aspects relevant for answering the research questions. The interviews were analysed following the steps of thematic analysis: becoming familiar with the data, generating initial codes, searching for themes, reviewing themes, defining and naming themes and reporting.^21^, ^22^ The programme MAXQDA 2020 supported the analysis process.^26^

To enhance the trustworthiness of the study, researcher triangulation was applied: one author (J. D.) analysed all 21 interview transcripts while 14 of the 21 transcripts were also analysed by at least one or more of the other authors (K. D. G., W. P. or A. F.).^27^ The whole team of authors discussed the interim and final analyses to further increase the trustworthiness and to make sure that the final themes presented in this paper clearly reflected the interview data.^27^

In the inductive analysis and related discussions, we identified four types of patients, distinguished by whether or not patients expressed a need to participate and whether or not patients expressed they were able to participate in nursing documentation. Analysing data by identifying types is a practically applicable and proven method, for example, in research on patient involvement and engagement.^28^, ^29^

We further enhanced the trustworthiness of the study by ‘peer debriefing’.^27^ This implied that we discussed a draft of this paper, also including the results sections, in an academic meeting with a group of peer researchers who were not involved in the study. Based on this peer debriefing, some small adjustments were made in the draft paper, in particular to write down the findings even more clearly.

Furthermore, we have provided descriptions of the setting and patient characteristics to help others judge the transferability of the results to other situations. In addition, the study is reported according to the ‘Standards for reporting qualitative research’ to boost the dependability of the study.^30^

### 2.5 Ethical considerations

The study protocol was approved by the Medical Research Ethics Committee of the Amsterdam University Medical Centre (file number 2019‐026). Patients signed a written informed consent form before the face‐to‐face interviews. Patients who were interviewed by phone provided their verbal, recorded informed consent.

The first author (K. D. G.) confirmed that all patient identifiers were removed from the transcripts so that the patients are not identifiable and cannot be identified through the details in their stories. The audio recordings were deleted as soon as the interviews had been transcribed.

## 3 RESULTS

### 3.1 Patient characteristics

In total, 21 patients took part in the study, of whom 15 were female (Table 2). The interviewed patients were between 24 and 88 years old and lived in various regions across the Netherlands. Over half of the participants (*n* = 13) received personal nursing care only (e.g., help with showering), while some patients received technical nursing care (e.g., care for a tracheostomy tube) or a combination (e.g., help with washing and infusion therapy).

**Table 2:** Characteristics of the patients (*n* = 21)

Characteristics	*N*	Missing
Age (mean; range)	69; 24–88	2
Gender		–
Male	6	
Female	15	
Educational level		2
Low	7	
Medium	8	
High	4	
Kind of home care		2
Personal nursing care	13	
Technical nursing care	5	
Combination of personal and technical nursing care	1	

### 3.2 Typology with four patient types

The analysis process resulted in the identification of a typology with four different patient types based on the individual patient's perceived need and ability to participate in electronic nursing documentation. The four types are illustrated by the following case narratives, which use fictitious names and are a composite of information on multiple patients.

*High need, high ability*: Mrs Peters is 35 years old, has a high educational level, and she receives home care every day for connecting her total parenteral nutrition. She actively participates in her own care and talks with nurses about the information they document in the electronic health record. When she has a check‐up with her physician, she brings along her iPad to show the observations documented by the nurses.

*High need, low ability*: Mrs De Boer is 73 years old, has a medium educational level, and she receives help with washing and dressing and for negative pressure wound therapy. She is interested in the nurses' observations during care moments. Yet most nurses do not tell her what is documented and she cannot read the information in the health record since she does not have a digital device and therefore she has no access to the electronic patient portal.

*Low need, high ability*: Mr Dijkstra, aged 62 with a low educational level, receives home care for the application of eczema ointment. He has full confidence in the nurses, who have been helping him for a number of years now. He feels no need to read or talk about the nurses' documentation. He has the ability to access the electronic patient portal on his iPad, but he has never looked at it.

*Low need, low ability*: Mrs Visser, aged 84 with a medium educational level, receives home care every day for putting on and taking off her compression stockings. The care she receives has been the same for quite some years now. She has no interest in what nurses document in her electronic health record. Besides, she does not own a digital device and she feels no need to buy a device to get access to her health record.

Even though a patient's position in the typology was not set in stone, participants could broadly be divided into these types. Eight patients were classified as ‘high need, high ability’. Four patients were assigned to each of the types ‘high need, low ability’ and ‘low need, high ability’. Lastly, five patients were classified as ‘low need, low ability’.

In general, we noticed some differences in the characteristics of the patients in the four types. Younger or more highly educated patients who received technical nursing care tended to fall in the type ‘high need, high ability’, whereas most elderly patients or less educated patients who received personal nursing care were assigned to the type ‘low need, low ability’.

### 3.3 Need to participate

The typology illustrated that the interviewed home‐care patients differed in their perceived need for participation in electronic nursing documentation. Virtually all patients in the types ‘high need, high ability’ and ‘high need, low ability’ stated some kind of personal interest as a reason for their need to participate. According to these patients, participation provided them with relevant information about their health situation and gave them insights into the nurses' assessment of their health situation. At the same time, some patients believed that the information in the nurses' documentation could be of interest to other healthcare professionals.

*Well, if I've gone downhill a bit, I would want to know how they interpret that. (…) That would give me some information about myself and that would be a kind of sign that I should contact my neurologist or my Parkinson's specialist*. (Patient 19)

Patients who were classified as ‘high need, high ability’ indicated additional reasons for their need for participation. For instance, participation gave these patients opportunities to correct the nurses' documentation if they disagreed or if they found the documentation to be incomplete. Additionally, these patients saw the benefits of accurate nursing documentation. They noticed that nurses were well aware of their situation after reading the health records. As a result, the patients did not have to explain their situation repeatedly to different nurses. This was particularly important for patients whose health situation was not stable and for patients who received complex technical nursing care.

*They ask whether anything's wrong, for instance to do with my health, and then I tell them that and I know they'll put that in the report. So the next one who comes along knows all about my situation, and I really like that*. (Patient 18)

In contrast to patients who felt a need to participate in nursing documentation, several patients said they felt no need for participation. These patients were classified in the types ‘low need, high ability’ and ‘low need, low ability’. They often said they had complete trust in nurses' documentation about what is important for their care, giving this as a reason for not feeling a need to participate. Furthermore, patients explained they did not want to be seen as meddling. Some patients felt that nursing documentation was more important for nurses than for themselves.

*When they get here, they open up their laptop and take a look first at what's been written there and all the things the person before them did. (…) I don't need to check that. That's how it works and they don't need to tell me exactly what everyone's written down; I don't need to know all that. (…) As I always say, they're the experts, not me*. (Patient 7)

Additionally, some patients felt less need to participate since they had no personal interest in nursing documentation. This was mostly indicated by patients who belonged to the type ‘low need, high ability’. Particularly in situations where nursing care was not complex, patients did not see any reason for participation. Yet patients stated that their need for participation did change over time, depending on their situation.

*Well, there wasn't that much to report and I was there myself so I don't really see the need. It's all so simple. Look, the hospital reports are a different matter—I'd like to read them again sometime*. (Patient 17)

### 3.4 Ability to participate

Patients not only varied in whether or not they felt a need to participate but they also differed in their ability to participate in electronic nursing documentation. Most patients who indicated being able to participate said they could read the documentation through the electronic patient portal. This applied to patients assigned to the types ‘high need, high ability’ and ‘low need, high ability’, and especially to patients who were young or middle‐aged. Some patients explained that electronic documentation had improved their ability to participate since electronic devices were easier to handle compared to paper‐based files.

*I control my computer digitally by my eye movements, so now I'm also scrolling through the patient portal. (…) I love it because now I can just look it all up on the computer*. (Patient 11)

Patients' ability to participate increased if nurses verbally guided them through documentation, during or directly after care. Several patients told that they felt encouraged to reflect on the documented information. This was mentioned by patients in both the types ‘high need, high ability’ and ‘low need, high ability’.

While some patients felt sufficiently able to participate, others felt less able to participate in the documentation. Most of these patients were of advanced age and belonged to the type ‘low need, low ability’. They said that they did not have any electronic devices or they lacked the digital skills to use electronic devices. These patients were therefore not able to read the documentation.

*I have got those things, those computers, but I don't understand them. (…) There's lots of things I can't do on the computer and then I think they should sort it out—that's fine by me*. (Patient 19)

Patients in the type ‘high need, low ability’ mentioned other reasons why they felt less able to participate, including limitations in the usability of the electronic patient portal. An example was not receiving responses to their messages. Moreover, two patients said that it was not possible to supplement or correct the nurses' documentation via the electronic patient portal.

*That system is the problem. (…) Once, they wrote that I was angry (…) and it's not possible to delete that so it still says I got angry even though I didn't. Yes, I did find that annoying, actually*. (Patient 14)

Another barrier indicated by patients in the ‘high need, low ability’ type concerned the nurses' working methods. Several patients felt that some nurses spend insufficient time guiding them through the documentation. Besides, some patients felt they had no opportunity to participate if nurses carried out their documentation outside of their homes.

*Not all of them. One takes the time to document it and reads it out too. But most do their documentation in the car*. (Patient 9)

Lastly, participants of the types ‘high need, low ability’ and ‘low need, low ability’ often explained that physical disabilities limited their ability to participate. Patients indicated they were either too sick or worn out, or lacked the concentration to actively participate in the documentation.

*I don't look at it. (…) Well, I've been pretty poorly. And then that kind of thing basically gets less of a priority at a time like that*. (Patient 4)

In some of these cases, patients mentioned that a spouse or another family caregiver stepped in. Yet others described not wanting to burden their family caregiver by asking them to participate in the documentation.

### 3.5 Ways to participate

We also asked patients about the ways in which they might ideally participate in electronic nursing documentation. Virtually all participants in the two types with a need for participation indicated that they preferred verbal communication with nurses about the documentation.

Interviewed patients who used an electronic patient portal also preferred to read the documentation via these portals. This was especially the case for patients belonging to the type ‘high need, high ability’. Besides, several patients talked positively about the possibility for family caregivers to read the documentation via the portal. These patients would then discuss the documentation together with their family caregivers. Mostly, patients of the type ‘high need, low ability’ mentioned this way of participation.

Regarding which parts of the documentation patients particularly preferred to participate in, these were the parts related to the performing and evaluation of nursing interventions.

*Simultaneously she tells me what she writes down about the care that she has provided and what she has noticed during this care moment. For instance, a small wound on the leg to which she has stuck a plaster*. (Patient 12)

Participation in the documentation of the nursing assessment, diagnosis and care planning seemed to be less of a priority for most patients. It is interesting to note that some patients did not even know what was documented in these parts of the documentation.

*I believe I've got it written down, in that folder. I must have read that sheet of paper but if you ask me to tell you what it was about, well, I don't remember much. (…) That's their thing*. (Patient 2)

## 4 DISCUSSION AND CONCLUSION

### 4.1 Discussion

In this study, a typology was identified with the following patient types: ‘high need, high ability’, ‘high need, low ability’, ‘low need, high ability’ and ‘low need, low ability’. The typology showed that patients differ in their perceived need and ability to participate in electronic nursing documentation.

A number of patients perceived their participation in nursing documentation to be important (those of the types ‘high need, high ability’ and ‘high need, low ability’). These patients had an interest in the documented information about their own health, the nurses' observations and the nurses' views towards their health and care needs. This finding is in line with previous research on patient participation and shared decision‐making,^4^, ^5^, ^6^, ^7^ as well as with current legislation and professional guidelines that support patient participation, for example, by stating that patients must have access to the documentation about their health and care.^24^, ^25^, ^31^, ^32^, ^33^

However, other patients (of the types ‘low need, high ability’ and ‘low need, low ability’) said they felt no need for participation. Some of these patients explained this lack of interest by stating that the nursing care they received was not complex and therefore the nursing documentation was not significant for them. Yet, other patients who did not feel a need to participate, explained that they considered participation to be a burden. These patients should not be pressured to participate in documentation, given that would be contrasting with the principle of need‐driven care, as was also indicated in previous research.^34^, ^35^, ^36^, ^37^ Nevertheless, in a qualitative interview study nurses indicated they still could involve patients to some extent, via verbal communication about the nursing care.^18^

At the same time, patients in the type ‘high need, low ability’ indicated that although they felt a need for participation, they did not always feel able to participate, for instance, because they could not read the electronic nursing documentation. There seem to be opportunities to enhance patient participation in nursing documentation for patients of this type, as these patients pointed to the importance of nurses helping them to reflect on nurses' documentation. If nurses failed to meet these needs, patients felt less able to participate. The importance of support from care professionals in achieving patient participation in nurses' or physicians' documentation was also indicated in other studies.^10^, ^38^

Furthermore, the poor usability of electronic patient portals was mentioned as a barrier for participation in nursing documentation by patients who used these portals. This seems in accordance with findings from previous studies.^39^, ^40^ Some participants in our study were not able at all to access electronic patient portals since they had no electronic devices or lacked the digital skills to use such devices. These patients were virtually all of advanced age, which is consistent with previous studies pointing to the limited use and usability of electronic patient portals for elderly persons.^41^, ^42^

In addition, previous studies indicated that low health literacy is associated with limited abilities to get access to information in electronic health records, for example, through patient portals.^43^, ^44^ Health literacy concerns the individual's cognitive and social skills that determine the ability to gain access, understand and use the information to promote one's own health.^45^ Furthermore, previous studies indicated that a low educational level and old age (both determinants of low health literacy) were related to a limited ability to access and understand health information.^46^, ^47^ In our study patients with a low educational level and/or advanced age also seemed to have less needs and abilities for participation in documentation, compared to younger or more highly educated patients.

In addition, some patients highlighted that their spouse or another family caregiver participated in nursing documentation, for example, by reading along and communicating with the nurses through the electronic patient portals. While patients were positive about this involvement of their family caregivers, they also told that the family caregiver's contribution to what is documented is limited. This is in line with a review on family engagement in electronic health records of hospital patients.^9^ This review reported that the participation of family caregivers in documentation rarely extended updating the patient information in electronic health records.^9^

Regarding our study, the fact that some of the interviews were conducted during the COVID‐19 pandemic needs to be taken into account. At that time there were restrictive measures for community nurses who only could visit a patient at home, when phone or online consultations were not appropriate. Yet we had no indications from the interviews that the pandemic and restrictive measures influenced patients' experiences with participation in nursing documentation.

Furthermore, it must be taken into account that we did not include participants who did not speak Dutch (which is relatively often seen in older patients with a non‐western migration background). Therefore, the results of this study are not transferable to non‐Dutch‐speaking people.

### 4.2 Conclusion

Home‐care patients differ in their need for participation in electronic nursing documentation. If patients perceive a need to participate, this is mostly based on an interest in documented information about their own health, and because they see the benefits of accurate documentation. If patients do not feel a need to participate, this is because they have complete trust in the nurses or feel a lack of interest since the nursing documentation was not significant for them.

The ability to participate in electronic nursing documentation differs between patients as well. Some patients are less able to participate since they have no electronic devices or lack the digital skills to access electronic health records. In addition, lack of support from nurses, the poor usability of electronic patient portals and poor health also limit patients' ability to participate.

Home‐care patients who want to participate prefer verbal communication with the community nurses and reading the documentation as ways to participate in electronic nursing documentation.

### 4.3 Practice implications

Whereas some patients expressed a need for participation in electronic nursing documentation, others do not. Therefore nurses should tailor their approach in encouraging patient participation to each individual patient. Moreover, needs for participation can change over time. This implies that nurses should verify the needs of home‐care patients not once, but continuously. Furthermore, some patients reported that they felt unable to participate because of a lack of support from nurses, for example, in reflecting on the documented information. Since most patients prefer verbal, direct communication about the content of the documentation, nurses should devote sufficient time to this. However, this can be challenging since community nurses cited time pressure as a barrier to achieving patient participation.^18^ Yet, patient participation might eventually save nurses time: if a patient participates in the documentation, there will be a greater chance of shared decision‐making about the care. This will ultimately lead to appropriate care that best suits patients' needs and which may be also efficient and time‐saving.

Lastly, a comment regarding the fact that we only had Dutch‐speaking patients in the sample. It is likely that the ability to participate in documentation will be limited if the patient cannot read the language used in the health records. In those cases, a patient will often be dependent on the translation by a family caregiver who does speak the language. This means that a community nurse will have to pay extra attention to communication about the documentation through the translating relative.

## CONFLICTS OF INTEREST

The authors declare no conflicts of interest.

## Data Availability

The anonymous transcripts that support the findings of this study are available from the corresponding author upon reasonable request that does not contravene the informed consent forms signed by the participants.
